# LncRNA MALAT1 was regulated by HPV16 E7 independently of pRB in cervical cancer cells

**DOI:** 10.7150/jca.61194

**Published:** 2021-08-28

**Authors:** Ting Wang, Wei Zhang, Wenbin Huang, Zichun Hua, Shufeng Li

**Affiliations:** 1 Key Laboratory of Developmental Genes and Human Disease in Ministry of Education, Department of Biochemistry and Molecular Biology, Medical School of Southeast University, Nanjing, 210009, China.; 2 Department of Pathology, Nanjing First Hospital, Nanjing Medical University, Nanjing 210006, China.; 3 The State Key Laboratory of Pharmaceutical Biotechnology, School of Life Sciences, Nanjing University, Nanjing, Jiangsu 210046.; 4 Changzhou High-Tech Research Institute of Nanjing University and Jiangsu Target Pharma Laboratories Inc., Changzhou, Jiangsu 213164, P.R. China.

**Keywords:** MALAT1, HPV16 E7, SP1, cervical cancer, transcription

## Abstract

High-risk human papillomavirus (HPV) infection was one of the first step in the process of carcinogenesis in cervical cancers. The expression of viral oncoprotein E7 was essential in this process by inactivating the tumor suppressor proteins RB, in addition to interacting with other host proteins. LncRNA MALAT1 was found to be altered in human cervical cancer tissues, suggesting an important role in tumorigenesis. Moreover, MALAT1 was also overexpressed in HPV16 positive cervical cancer cell lines in an HPV16 E7 dependent manner. To explore the mechanism of E7 involved in MALAT1 up-regulation, the deletion mutant E7∆N and E7∆C were constructed to test the functional domain of E7 for MALAT1 regulation. ChIP, EMSA and UV crosslink were performed to detect the interaction between E7 and MALAT1 promoter. E7 and E7∆N mutant were observed that could bind with MALAT1 promoter directly and interacted with SP1 to form triple complex. E7-SP1 interaction contributed to the transcription activation of MALAT1 promoter. E7 and E7∆N also could promote cell proliferation, invasion, and migration, and the stimulating effect could be reversed by siMALAT1. Here we showed that HPV16 E7 as well as E7∆N could associate with SP1 and bound directly to MALAT1 promoter *in vitro* and *in vivo*. This function way of E7 was independent of pRB in cervical cancer cells. To our knowledge, this was the first reported function model for E7 as transcription activator to directly bind to the target promoter.

## Introduction

HPV infection was a critical step in the development of cervical cancer. It was shown that HPV genetic material can be found in almost all cervical cancer tissue 1. For hr-HPV16, the major oncogenic proteins that mediate cell transformation were E6 and E7. It was believed that E7 exerted cellular immortalization and transformation by interfering with the functions of the cellular tumor suppressor proteins RB, mediating the uncontrolled cell proliferation 2. In addition to RB, a large number of studies have shown that it has the capacity to interact with a variety of cellular proteins involved in the regulation of cell cycle and transcriptional control. E7 oncoprotein can bind to c-Fos and upregulated AP-1 activity 3, it was also showed promoter activation and repression capabilities 4-8. Although the underlying mechanisms behind these functions were still poorly understood, it was already known that E7 can interact with some of the basic elements of the transcriptional machinery, including TAF110 and TATA box binding protein (TBP) 9, 10.

Metastasis-associated lung adenocarcinoma transcript 1 (MALAT1) was a broadly expressed lncRNA involved in many aspects of cellular processes. MALAT1 was first described to be associated with metastasis of lung tumors 11, it was also shown to be associated with the proliferation and metastasis of cervical cancer 12. Elevated levels of MALAT1 were observed in cervical cancer cells, clinical samples and exosomes of cervical cancer 13. It was noticed that MALAT1 level in cervical cancer was associated with HPV infection and E7 level 14. For the previously observed correlation between MALAT1 and cervical cancer development, MALAT1 was proposed to be a potential diagnostic marker 15. However, it was still unknown if MALAT1 alterations were caused by HPV infection during cervical carcinogenesis. The purpose of this study was to investigate HPV16 E7 on the expression of lncRNA MALAT1 in cervical cancer cells.

In the current research, by methods of ChIP, EMSA and UV crosslink, both E7FL and E7ΔN mutant which abrogates binding to RB, were observed that could bind with MALAT1 promoter directly and interacted with SP1 to form ternary complex. HPV16 E7 was found to affect the transcription level of MALAT1 via SP1 driven promoter sites. These findings reveal a novel mechanism involved in transcriptional activation by E7 protein in a retinoblastoma protein-independent manner. To our knowledge, this was the first reported function model for E7 to directly bind to DNA and promoted target gene transcription.

## Materials and Methods

### Cell lines, plasmids and antibodies

The human cervical cancer cell lines: SiHa and Caski (HPV16 positive cervical cancer cell lines), HeLa (HPV18 positive cervical cancer cell lines), C33A (HPV negative cervical cancer cell), and HEK-293T were from ATCC.

E7 VHH2 overexpressing plasmid pCMV-E7 VHH2 and SP1 overexpression plasmid pCMV-flag-SP1 was constructed by our lab previously 16, 17. pGM-SP1-Luc (SP1 luciferase reporter plasmid containing SP1-responsive element) was from Yeasen Company (China).

pCMV-myc-E7 expressed wild type E7 (E7 Full length 1-98 aa), while pCMV-myc-E7ΔN and pCMV-myc-E7ΔC expressed truncated E7 (aa 28-98 for E7ΔN, 1-58 aa for E7ΔC). These E7 and truncated E7 expressing plasmids were constructed by inserting full length or truncated cDNA of E7 into pCMV-myc plasmid.

For SP1-C expressing plasmid construction, SP1-C coding fragment (expressing 611-785 aa of truncated SP1 containing zinc finger protein motif, by which it binds directly to DNA) was synthesized and cloned into pet-32a plasmid and recombinant plasmid pet-32a-SP1-C was transformed in E.coli, expressed recombinant SP1-C fusion protein obtained His and Trx tag.

His-E7 expression plasmid were described previously 16. Briefly, the synthetic HPV16 E7 cDNA was ligated into psumo-mut vector and transformed into E. coli cells. The His and sumo-tagged E7 fusion protein was expressed in E.coli BL21 (DE3) cells with IPTG induction.

Antibody anti-pRB was from Affinity Biosciences (China); anti-HA was ordered from Beyotime Biotechnology (China); and anti-E7 was from Abcam (UK); anti-flag, anti-myc and anti-actin were purchased in Proteintech company (China).

### RNA extraction and real-time PCR

RNA isolation was performed using TRIzol reagent. Then cDNA was synthesized. PCR and RT-qPCR were performed. Primer sequences are available upon request. The 2^-∆∆Ct^ method was used to calculate the relative quantification of gene expression levels.

### Dual-luciferase assay for promoter activity

MALAT1 promoter luciferase constructs (pGL-319, pGL-288, pGL-288m and pGL-247) were previously reported 18. Cells were plated on plates, cultured overnight, and transfected using lipofectamine 2000, pRL-TK control vector encoding Renilla luciferase was used for co-transfection for normalization of luciferase activity. After transfection for 48 h, cells were lysed and luciferase activity was detected.

### Chromatin immunoprecipitation (ChIP)

ChIP assay was performed as previously described 18. After transfection for 48 h, cells were crosslinked with formaldehyde and then lysed. After sonication and incubation with anti-myc overnight, followed by immunoprecipitation with protein G agarose beads. Enriched DNA was extracted and PCR was performed. Precipitated MALAT1 promoter DNA was amplified.

### Electrophoretic mobility shift assay (EMSA)

Expression and purification of recombinant HPV16 E7 proteins in E.coli were described previously 16. Meanwhile nuclear extract from pCMV-myc-E7FL or pCMV-myc-E7ΔN transfected cells were also prepared. Synthetic complementary oligonucleotides containing the SP1-binding site of MALAT1 promoter were 5′-biotinylated and annealed. The wild-type sequences of the oligonucleotides used were as follow (the SP1-binding site were underlined): probe1 sequence: 5′-CAGGC GTTAGGGCGGGGCGCGCGTGC-3′, probe 2 sequence: 5'-AGCTGCCACCCCGCCCCCATGCCAT-3'. The mutant probe sequence: 5′-GGCAC AGGCG TTAΔ8GGCGCGCGTGCGCAG-3′. DNA-protein-binding assays were carried out, and detected using HRP-conjugated streptavidin (Chemiluminescence EMSA kit).

### UV crosslinking for protein purified from E.coli

Purified protein was mixed with synthetic complementary oligonucleotides probe. Binding buffer was added and incubated for 20 minutes. The samples were irradiated at 254 nm for 30 minutes on ice, then boiled for 3 minutes, electrophoresed on 12% SDS-PAGE and detected using standard western blot analysis.

### UV crosslinking for total lysate of cells transfected with plasmid

After transfection 48 h, cells were lysed and mixed with synthetic complementary oligonucleotides probe which contained the Sp1-binding site of MALAT1 promoter. The mixtures were irradiated, followed by the incubation with the indicated antibody at 4 °C for 12 h, and then precipitated with Protein G agarose beads. After washing and boiling, the retrieved proteins were detected using standard western blot analysis.

### Immunoprecipitation and Western Analysis

Cells were co-transfected pCMV-flag-SP1 with pCMV-myc-E7FL or pCMV-myc-E7ΔN or control pCMV-myc. After 48h, whole cell lysates were prepared and incubated with anti-myc mAbs for 12h. Immune complexes were isolated with protein G agarose and washed. Bound proteins were analyzed by SDS-PAGE followed by western blotting. For western blot analysis, separated protein on gel were transferred to PVDF membrane, and detected with the indicated primary antibody, then followed by incubation with HRP-conjugated secondary antibody, finally immunoreactive signals were developed.

### Cell proliferation assay

Cell proliferation was determined by the Cell Counting Kit-8 according to manufacturer's manual. Briefly, 2 × 10^3^ C33A cells were seeded in 96-well plates per well. After transfection indicated time, the medium was changed and 10 µL of CCK-8 was added to each well and incubated for another 3 hours, the absorbance value at 450 nm was measured.

### Colony Forming Assay

Forty-eight hours after transfection, C33A cells were counted and seeded into a 6-well dish and cultured to allow colony formation. After incubation for 12 days, cells were washed and fixed, then stained with Gimesa. Visible colonies were counted. All experiments were performed in triplicate.

### Wound-healing assay

Wound-healing assay was carried out as previously described 17. Briefly, after transfection, C33A cell growth was allowed to continue until confluence was reached. Then the cell layer was scratched by a 200 μl pipette tip and dislodged cells were washed away. The average extent of wound closure was quantified. Cell culture continued 24 h, and the area covered by migrated cells were calculated.

### Transwell assays

Transwell assays were performed using 24-well plates with 8-μm filters (Corning, USA). 12 hours after transfection of the corresponding plasmids, C33A cells were treated with serum starvation. 1×10^5^ cells in 200 μl of serum-free medium were placed in the upper chamber and 500 μl 18% FBS medium was injected in the lower chamber. After 48 hours of incubation, the migrant cells attached to the lower surface were fixed, photographed and quantitatively processed, and each experiment was repeated three times.

### Cervical tissues

All cervical tissues were obtained from Nanjing First Hospital. Hematoxylin-eosin (H&E) staining was carried out for pathological diagnosis. DNA extraction and PCR were performed for HPV detection. The Ethics Committee of Nanjing First Hospital approved this study and informed consent from each participant was obtained.

### siRNA

The siRNA sequence of si-E7-1 and si-E7-2 were from previous report 19, 20. siRNA targeting RB (si-RB-1 and si-RB-2) were from the following references 21, 22. siMALAT1 was described in our previous report 23. All these siRNA were synthesized in GenePharma Company, China.

### Statistical analysis

The data was presented as mean ±SD of triplicate experiments and analyzed using student's t test. Statistical analyses were performed with GraphPad Prism 7 and SPSS 18. P < 0.05 was considered to be significant.

## Results

### MALAT1 was upregulated in cervical tissues and cervical cancer cell lines infected with HPVs

**Figure 1:**
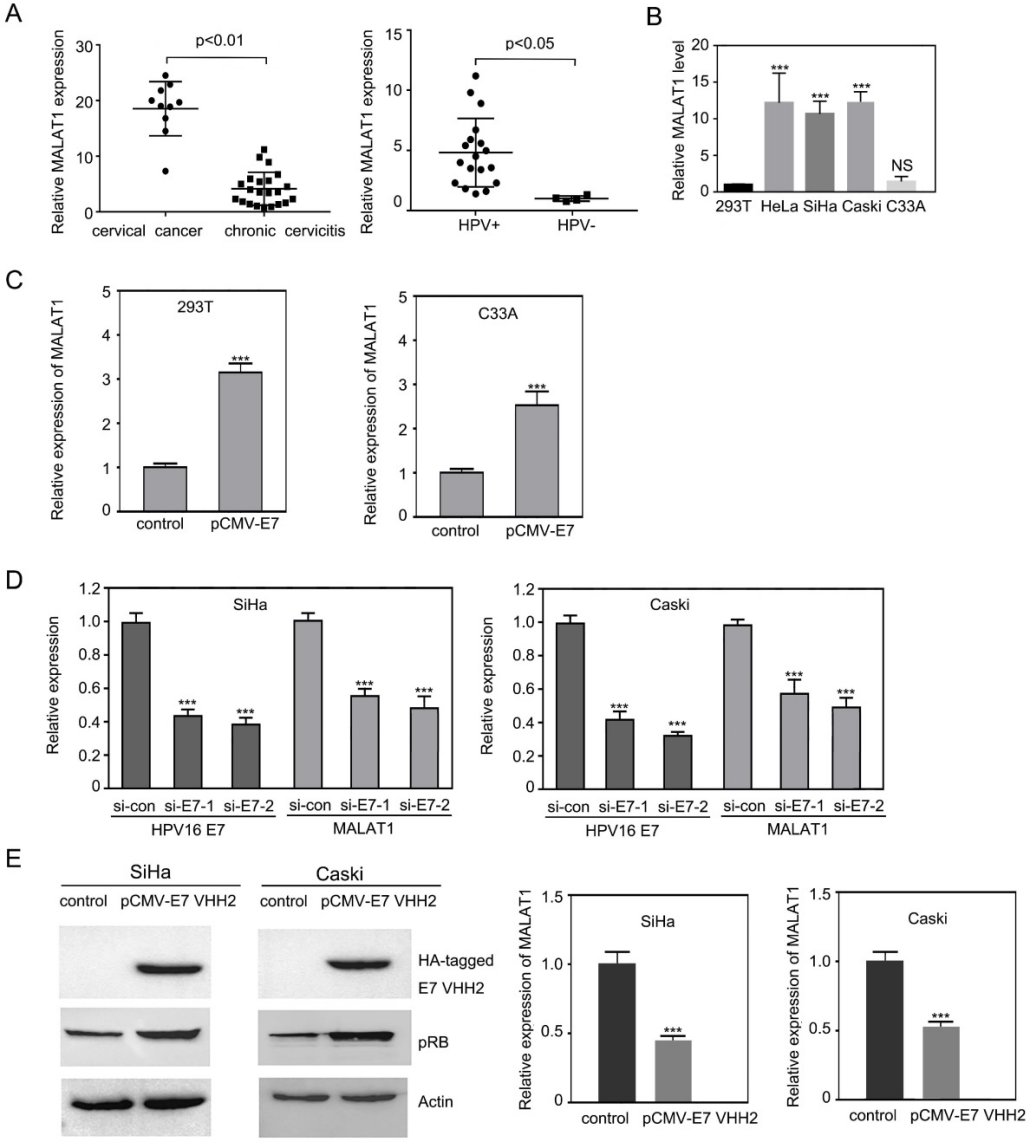
** MALAT1 was upregulated by HPV16 E7. A.** The level of MALAT1 RNA transcript were elevated in human CC and HPV positive cervical epithelia tissues.MALAT1 RNA levels in human cervical tissues were detected by RT-qPCR. **B.** By RT-qPCR, lncRNA MALAT1 was shown up-expressed in high-risk HPV infected cervical cancer cells HeLa (HPV18 positive), SiHa and Caski (HPV16 positive), compared with C33A (HPV negative) cells and 293T. **C.** Overexpression of HPV16 E7 upregulated MALAT1 when pCMV-E7 transfected 293T and C33A cells and RT-qPCR was performed. **D.** Depletion of HPV16 E7 with siRNA against E7 transfection significantly decreased MALAT1 level. **E.** With HPV16 E7 intrabody VHH2 expressing plasmid transfection in SiHa and Caski, pRB was increased, and MALAT1 RNA was decreased. Left: western blot. Right: RT-qPCR analysis. Similar results were obtained in at least three independent experiments. GAPDH mRNA was used to normalize the qRT-PCR analyses. *** p ≤ 0.001.

In order to confirm the correlation between MALAT1 with HPV infection in cervical cancer, MALAT1 RNA levels were examined using qRT-PCR in clinical specimen from 10 cervical epithelial squamous cell carcinoma samples with HPV+ and 22 chronic cervicitis samples (18 HPV+ and 4 HPV-). The levels of MALAT1 RNA in cervical epithelial squamous cell carcinoma were significantly higher when compared with the cancer-free tissue with chronic cervicitis (Figure 1A). In addition, MALAT1 levels were higher in HPV positive cervical tissues than those in HPV negative cervical tissues (Figure 1A), indicating that MALAT1 level was associated with HPV infection. MALAT1 RNA levels in three HPV positive cervical carcinoma cell lines (CaSki,SiHa and HeLa) were also examined. As shown in Figure 1B, higher expression of MALAT1 was observed in the three HPV+ CC cell lines when compared with non-HPV infected C33A cells and control 293T cell. This suggested that MALAT1 was involved with HPV infection and played an important role in tumorigenesis.

### Overexpression of HPV16 E7 increased the lncRNA MALAT1 while siRNA or intrabody against HPV16 E7 decreased MALAT1 level

Due to the role of HPV16 E7 in cervical lesion, it was further investigated that whether HPV16 E7 oncogene induced the upregulation of MALAT1.In 293T and C33A cells, pCMV-E7 was transfected. Overexpressed HPV16 E7 enhanced MALAT1 expression significantly (Figure 1C). To further confirm these findings, HPV16 E7 was knocked down in Caski and SiHa cells by two different siRNAs against HPV16 E7. After reduction of HPV16 E7, MALAT1 RNA level was down-regulated (Figure 1D). Based on our previous research 16, 24, intrabody anti-HPV16 E7 VHH2 presented the ability to counteract the activity of target E7 proteins. This anti-HPV16 E7 VHH2 was further used to evaluate its effect on the MALAT1 level. It was observed that pRB level was enhanced by E7 VHH2 expression indicating VHH2 blocked the activity of E7. Meanwhile, the level of MALAT1 was also noticed significantly downregulated. These data indicated that E7 was involved in the upregulation of MALAT1.

### MALAT1 expression was regulated by HPV16 E7 in a pRB-independent manner

**Figure 2:**
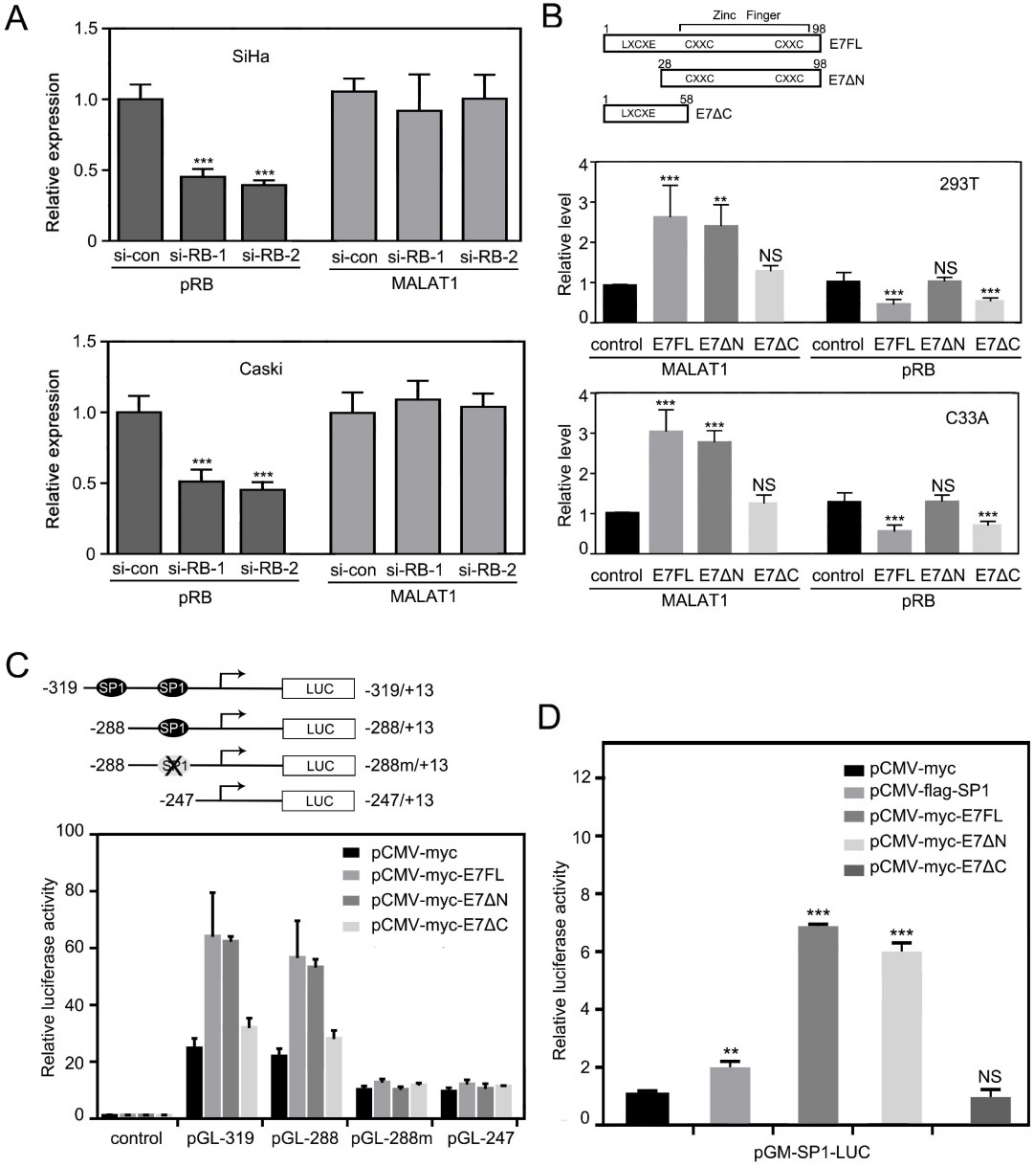
** MALAT1 expression was regulated by HPV16 E7 in pRB-independent manner. A.** RT-qPCR analysis of MALAT1 level and western analysis of pRB level in SiHa and Caski cells transfected with siRNA against RB. **B.** MALAT1 level was not correlated with pRB level. Upper panel: Schematic diagram of HPV16 E7 full-length construct E7FL, deletion mutant E7ΔN (28-98) and E7ΔC (1-58). LXCXE denotes the RB-binding motif; CXXC shows the positions of cysteine pairs that form the zinc finger. Lower panel: By inserting the E7FL, E7ΔN and E7ΔC into pCMV-myc vector, the plasmid expressing E7FL, E7ΔN and E7ΔC were constructed. HPV16 E7FL, E7ΔN and E7ΔC encoding plasmids were transfected into indicated cells for 48h, MALAT1 were detected by RT-qPCR. pRB level were detected by western blotting and analyzed with image J. **C.** SP1-binding sites was critical for E7 to stimulate MALAT1 promoter activation. Upper panel: Construction of promoter reporter plasmids for MALAT1 promoter activity with or without SP1 binding sites. The schematic diagram of the luciferase reporter constructs containing the promoter of the MALAT1. The two SP1 binding sites on the MALAT1 promoter were shown. The single SP1 binding site at the proximal end was mutated and constructed into a -288m/+13 fragment. Lower panel: E7FL and E7ΔN overexpression enhanced MALAT1 promoter activity. Overexpression of HPV16 E7 activated MALAT1 promoter activity through SP1-binding site. C33A cells were transiently co-transfected HPV16 E7FL, E7ΔN and E7ΔC expressing plasmids, or empty vector pCMV-myc along with the indicated luciferase reporter constructs together with the Renilla luciferase reporter plasmid. Forty-eight hours after transfection, firefly and Renilla luciferase activities were measured by dual-luciferase assay system. Data were shown as fold induction compared to the activity of cells transfected with the control pGL-4.17 vector. **D.** HPV16 E7FL and E7ΔN can activate SP1-mediated transcription. The luciferase intensity was analyzed in C33A cells by co-transfecting indicated plasmids and SP1-Luc reporter plasmid. After 48h of transfection, luciferase activity of SP1-LUC was analyzed. **, P<0.01; ***, P < 0.001.

It is well known that one of the major HPV E7 targets is the tumor suppressor pRB, which is involved in the regulation of cell cycle. Interestingly, when we knocked down pRB by using two different siRNAs, we observed that MALAT1 expression did not change (Figure 2A), suggesting a regulation by HPV16 E7 was not through pRB pathway.

HPV16 E7 protein was composed of 98 amino acids 10. It was reported that E7 could bind to retinoblastoma tumor repressor (RB) through its N terminal LXCXE motif, but LXCXE binding was not sufficient for E7 to induce proliferation 25. To further test the hypothesis that E7 regulating MALAT1 did not depend on RB pathway, expression plasmid of wild type E7 (E7FL) and deletion mutant (E7ΔN with LXCXE motif deleted, E7ΔC with Zinc finger motif deleted) were constructed (Figure 2B). Then constructed vectors were transfected in 293T cells and C33A cells, and then MALAT1 RNA level was measured by RT-qPCR following transfection. Meanwhile, pRB levels were also detected by western and analyzed. Results showed that although E7ΔN lost its activity to affecting pRB level, E7ΔN transfection still upregulated MALAT1 level as that of E7FL (Figure 2B). This data confirmed that HPV16 E7 regulated MALAT1 in pRB independent manner.

### HPV16 E7 regulates the level of MALAT1 via MALAT1 promoter

Based on above observation, it was supposed that HPV16 E7 maybe directly influence MALAT1 transcription. Therefore, whether MALAT1 promoter activity be promoted by HPV16 E7 was investigated next. In our previous research, MALAT1 promoter construct were cloned and two SP1 binding sites in the core promoter of MALAT1 were identified (Figure 2C). SP1 was found that played a critical role in MALAT1 transcription 18. To investigate the mechanism of E7 upregulating MALAT1, C33A cells were transiently co-transfected E7 FL expression plasmid or deletion mutants with the indicated MALAT1 promoter-luciferase constructs. It was observed that E7FL and E7ΔN promoted the MALAT1 promoter activity significantly (Figure 2C). In addition, it was suggested that E7 contributed to the MALAT1 transcription activation mainly through the -319/-247 region of MALAT1 promoter, meanwhile the results also indicated that after SP1 binding site mutated, the effect of E7 on MALAT1 promoter activity was lost (Figure 2C). Subsequently, in order to investigate the relationship between E7 and SP1 binding sites, C33A cells were transiently co-transfected SP1 luciferase reporter plasmid pGM-SP1-Luc with E7 or SP1 expressing plasmid. Overexpression of E7FL or E7ΔN resulted in a significant increase of promoter activity of SP1-Luc, and the stimulatory effect was even higher than that of SP1 overexpression (Figure 2D). These results indicated that HPV16 E7FL or E7ΔN upregulate MALAT1 transcription via the SP1-driven promoter.

### Wild-type E7 or E7ΔN bind directly to the MALAT1 promoter via the SP1 binding sites

**Figure 3:**
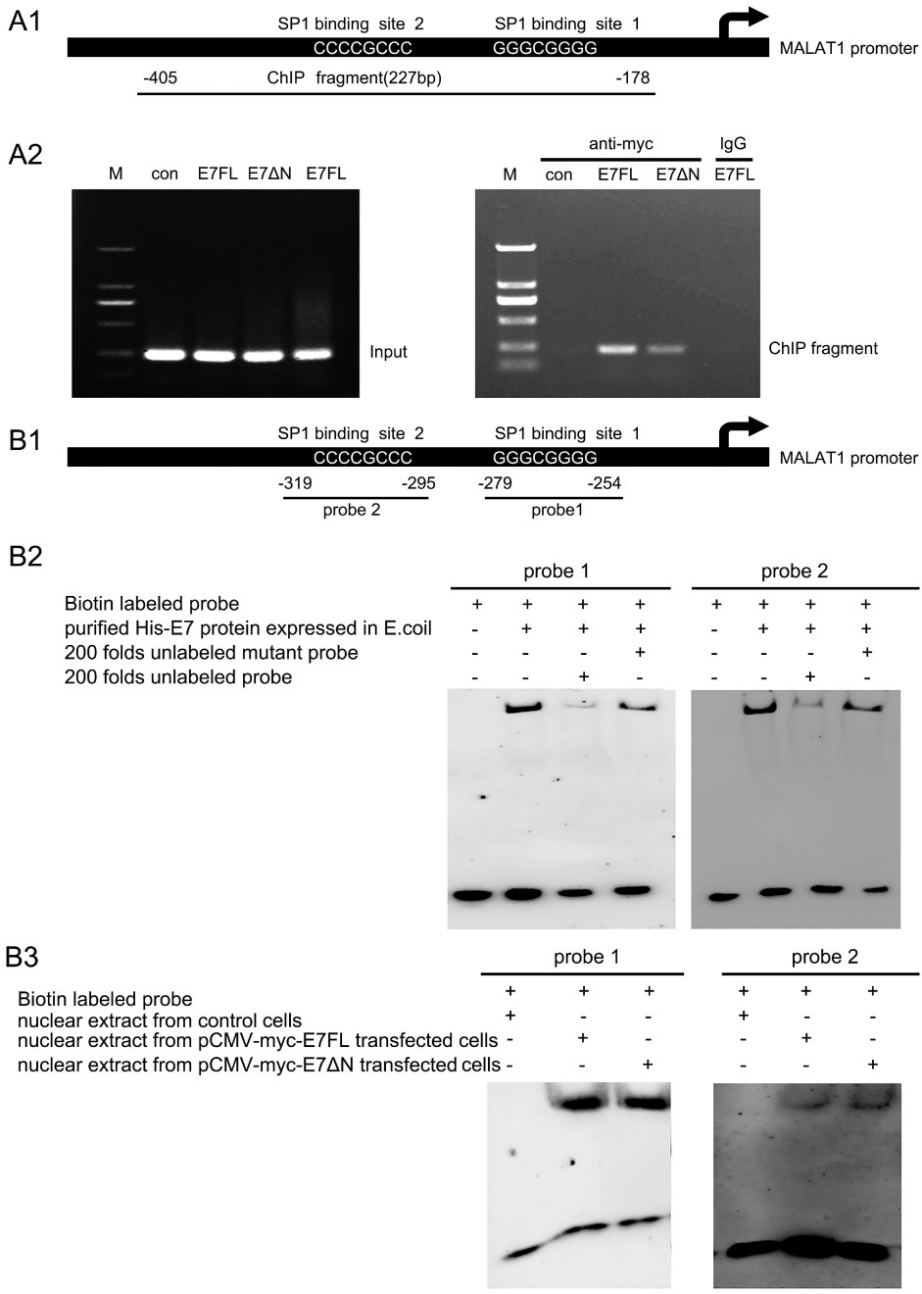
** HPV16 E7FL or E7ΔN bind directly to the SP1 binding sites of the MALAT1 promoter. A.** HPV16 E7 binds to the MALAT1 promoter region via zinc finger domain. **A1:** Schematic of the MALAT1-promoter showing the position of the SP1 binding sites and the locations of ChIP-PCR fragments. **A2:** ChIP-PCR results: Chromatin was prepared from C33A cells transfected with indicated plasmids. When chromatin from transfected cells was immunoprecipitated with anti-myc antibody, MALAT1 promoter DNA was amplified. **B.** HPV16 E7 directly binds to the GC box (SP1 binding sites) on the MALAT1 promoter via zinc finger domain. **B1:** Schematic of the MALAT1-promoter showed the position of the SP1 binding sites and the locations of two probes used for EMSA assay. **B2-B3:** EMSA analysis. **B2:** Purified HPV16 E7 protein from E.coli can bind with both probes, and this binding was blocked by excess unlabeled probe, whereas unlabeled mutant probes did not present the blocking effect. **B3:** Nuclear extract from transfected C33A cells was used to incubate with probes, both E7 and E7ΔN formed complex with probes.

To further address whether E7 could bind directly to the MALAT1 promoter, ChIP analysis was performed. The locations of ChIP-PCR fragments were shown in Figure 3A1. ChIP-PCR results were shown in Figure 3A2. After overexpression of wild-type E7 or E7ΔN in C33A cells, the cross-linked chromatin was prepared and were immunoprecipitated with control IgG or antibody anti-myc, the immunoprecipitated DNA were individually amplified with primer sets covering the MALAT1 promoter. As shown in the Figure 3A2, the PCR product of MALAT1 promoter was detected after pulling down E7 in E7-overexpressing C33A cells. This implies that E7 bound to the MALAT1 promoter. In addition, in E7ΔN overexpressing C33A cells, ChIP-PCR product was also detected, indicating that the C-terminal part of E7 was critical for the E7 binding to MALAT1 promoter.

To further determine whether binding of E7 or E7ΔN to the MALAT1 promoter was via the SP1 binding sites, EMSA assay were performed. The two probes used for EMSA assay covered the SP1 binding sites (the GC box), the location of them on the promoter were shown in Figure 3B1. The synthetic biotinylated probe containing the GC box of the MALAT1 promoter was incubated with purified HPV16 E7 protein from E.coli 16. EMSA result showed that both probe1 and probe2 bound with E7 protein, and this binding was inhibited by excess unlabeled probe, whereas unlabeled mutant probes did not present the inhibiting effect (Figure 3B2). To confirm that C33A cells overexpressed wild-type E7 or E7ΔN also could bind with probe1 or probe2, another EMSA were performed (Figure 3B3). Nuclear extracts from C33A transfected with pCMV-myc-E7FL or pCMV-myc-E7ΔN was used to incubate with probes, it was observed that both wild-type E7 and E7 mutant can form complex with the two probes. Above results suggested that HPV16 E7 directly bound to the GC box on the MALAT1 promoter.

### Wild-type HPV16 E7 or E7ΔN can interact with SP1 and form a complex with MALAT1 promoter

**Figure 4:**
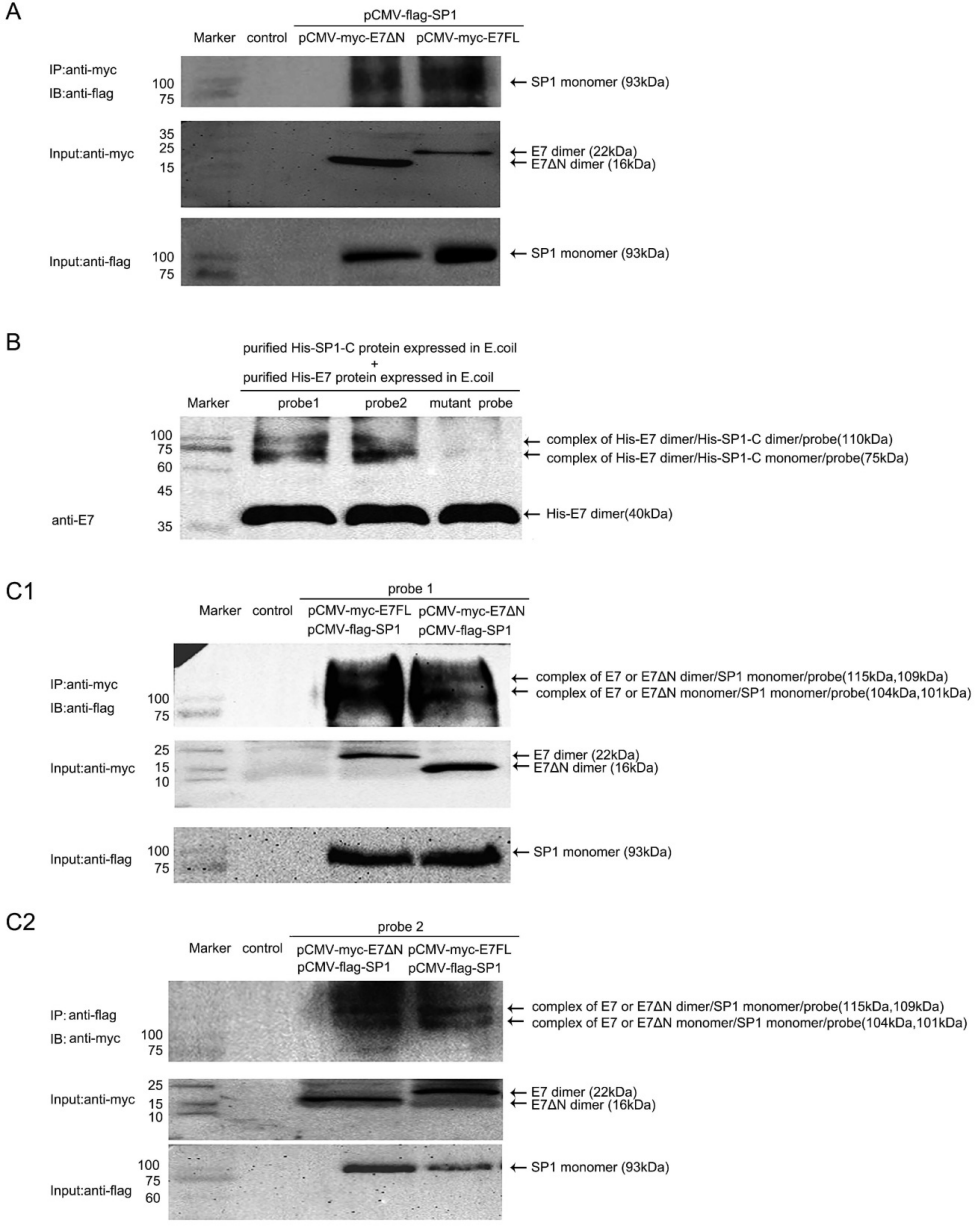
** E7FL or E7ΔN interacted with SP1 and formed complex with MALAT1 promoter. A.** Co-IP assay for Myc-E7 FL or Myc-E7ΔN and Flag-SP1. E7 or E7ΔN interacted with SP1 in C33A cells. Cells were co-transfected indicated plasmids, following anti-myc IP, coprecipitated SP1 was revealed by anti-flag immunoblotting (93 kDa). Input levels of myc-E7 (22 kDa) or myc-E7ΔN (16 kDa) and flag-SP1 (93 kDa) were also shown. **B.** UV crosslinking assay for protein purified from E.coli: His-E7 (40 kDa) and His-SP1-C (35 kDa) can form complexes with the MALAT1 promoter DNA *in vitro*. Arrows indicated the complexes formed (about 75 kDa and 110kDa), and the band of unbound His-E7 was also shown. No complexes band was observed when the mutant probe was used. **C.** UV crosslinking assay for total C33A cell lysates with indicated plasmid overexpression: Arrows indicated the complexes formed by probe, myc-E7FL/E7ΔN and flag-SP1 (about 104 kDa, 115kDa for the lane of myc-E7FL; 101 kDa, 109 kD for the lane of myc-E7ΔN).

Although E7 binding to the SP1 sites on the MALAT1 promoter was approved, then how the E7 and SP1 involved in the promoter activation? Specificity protein 1 (SP1) was one of the first mammalian transcription factors to be characterized. SP1 was a member of a family of proteins with highly related zinc-finger domains that bind to GC boxes in the regulatory regions of many housekeeping as well as tissue-specific genes 26. In our previous research, SP1 was found to bind with probe1 and played a critical role in regulating MALAT1 transcription 18. Here we further assess the relationship that E7 and SP1 together involved in the promoter activation with the condition that E7 was transiently overexpressed. Firstly, Co-IP assay was performed. C33A cells were co-transfected pCMV-flag-SP1 with pCMV-myc-E7FL or pCMV-myc-E7ΔN. The co-IP result showed that wild type HPV16E7 or E7ΔN interacted with SP1 in C33A cells (Figure 4A). Our results suggested that SP1 was a novel E7-binding protein that was potentially involved in the generation of human cancer. Secondly, it was investigated that whether E.coli expressed E7 and SP1 interacted with MALAT1 promoter and formed a ternary complex *in vitro*. Recombinant His-E7 or His-SP1-C was expressed and purified according to Li et al 16, 27. By UV crosslinking for protein purified from E.coli with probe, the purified His-E7 (40 kDa) and His-SP1-C proteins (35 kDa) formed complexes with the MALAT1 promoter probe DNA. As shown in Figure 4B, two bands of complexes were observed. It was speculated that the complexes were composed of SP1-C monomer or SP1-C dimer with E7 dimer protein and probe. Finally, it was investigated that overexpressed E7 and SP1 in C33A cells also could interact with MALAT1 promoter probes and form ternary complex. By UV crosslinking probe mixed with total lysates of C33A cells overexpressing myc-E7FL or myc-E7ΔN and flag-SP1, the results showed that myc-E7FL or myc-E7ΔN co-precipitated with flag-SP1 and form ternary complexes with probes. Two bands of complexes were observed (Figure 4C1 and 4C2), maybe because the E7 was in form of monomer or dimer. These results indicated stable ternary complexes were formed by E7, SP1 and promoter DNA probes. Meanwhile, the involvement of E7ΔN or His-SP1-C protein in complexes formation suggested that the carboxyl terminus of both E7 and SP1 contributed to the interaction.

### E7 FL and E7ΔN promote cell proliferation, invasion, and migration, which can be reversed by siMALAT1

**Figure 5:**
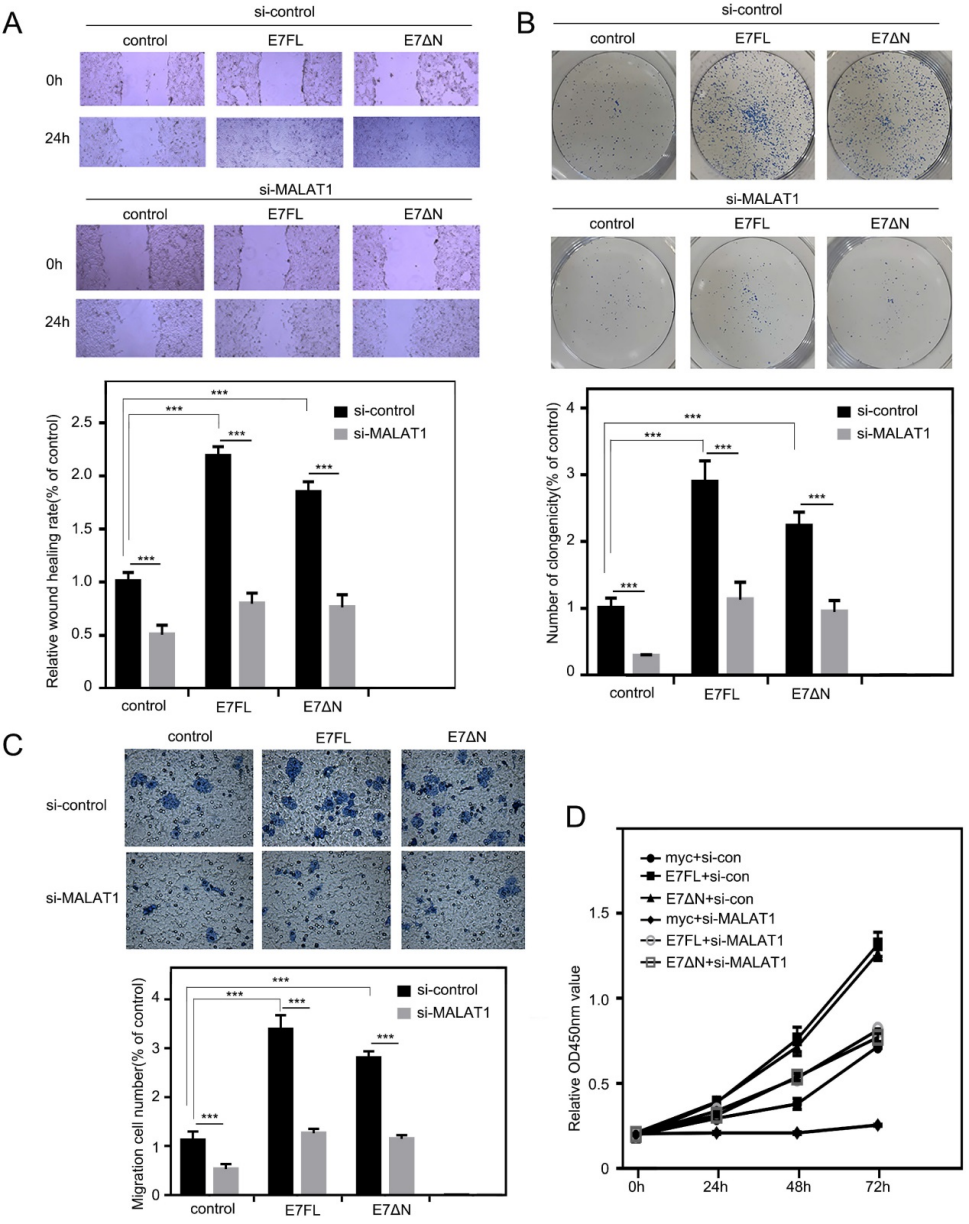
** E7 FL and E7ΔN promote cell proliferation, invasion, and migration, which can be reversed by siMALAT1. A.** Wound healing experiments were used to detect the effect of siMALA1 on motility of C33A cells with E7 FL or E7ΔN overexpression. C33A cells were transfected with indicated plasmid, and then wound-healing scratch motility assays were performed. Cell invasion was assessed at 0 and 24 h. Representative images were shown in upper panel. Percentages of wound healing rates were shown in lower panel (***, P < 0.001). **B.** Colony-forming assay. Upper: C33A cells were transfected the plasmids as indicated. Lower: Statistical analysis of clonogenicity. The number of clonogenicity was presented as the mean ± SD of three different experiments (***, P < 0.001). **C.** Knockdown of MALAT1 inhibited the migration of C33A cells overexpressing E7FL and E7ΔN. HPV16E7FL or E7ΔN were cotransfected with siMALAT1, after 48h, migrated cells were measured as shown in upper panel. Quantitative assessment of the number of cells migrated to the lower chamber was shown in bottom. **D.** The proliferation of C33A cells which were transfected indicated plasmids, were tested by CCK8 assay after transfection indicated time.

Our above results indicated that E7 protein as well as E7ΔN regulated the level of MALAT1 in cervical cancer cells via SP1-driven promoter sites. Then, the effect of E7 upregulated MALAT1 on cell proliferation, invasion and migration were examined. As shown in Figure 5A, the overexpression of E7FL or E7ΔN in C33A cells resulted in more cells migrated into wound area as compared with cells transfected with control vector. In addition, in colony formation assay, enhanced effect by E7FL or E7ΔN were also observed (Figure 5B). In transwell assay, E7FL and E7ΔN also promoted migration (Figure 5C). Finally, CCK-8 assays indicated that E7FL or E7ΔN both remarkably increased C33A cells growth (Figure 5D), and all above promotion effect could be reversed by siMALAT1. Collectively, these data indicated that HPV16 E7, particularly the carboxyl terminus of E7, contributed to upregulated MALAT1 through SP1-driven MALAT1 promoter.

## Discussion

It was clear that E7 plays a key role in the early carcinogenesis of high risk HPV infection 28. HPV16 E7 was known to bind to tumor suppressor protein RB and promoted RB degradation 29. However, other functions of E7 were also necessary for the transformation of infected cells 30. For example, a pRB binding-defective mutant of E7 can still immortalize primary human keratinocytes in cooperation with E6 31.

In our research, overexpression of HPV16 E7FL or E7ΔN both promoted cell proliferation, migration and invasion through increasing MALAT1 transcription. Subsequent ChIP and EMSA results showed that E7 as well as E7ΔN both bound directly to the MALAT1 promoter *in vitro* and *in vivo*. Our results revealed the first time that the HPV16 E7 protein directly bound to the GC box of DNA promoter. This interaction was found to take place through residues in the carboxyl terminal part of E7. Based on the fact that E7ΔN contain conserved zinc-finger domain 32, and it was ever supposed that zinc finger domain were suitable for binding GC rich nucleic acid fragments 33, therefore we postulated that the zinc finger domain of E7 was responsible for the GC-box DNA binding. Our result was also consistent with previous hypothesis that E7 was suggested to interact with nucleic acids 32. However, until now there were no any article reporting evidence about E7 directly binding to DNA. For the best studied activities of E7 proteins were related to the ability to interact with target cellular proteins, our results provided evidence that E7 directly bind to promoter DNA and revealed a novel pathway for E7 transcriptional activation function.

**Figure 6:**
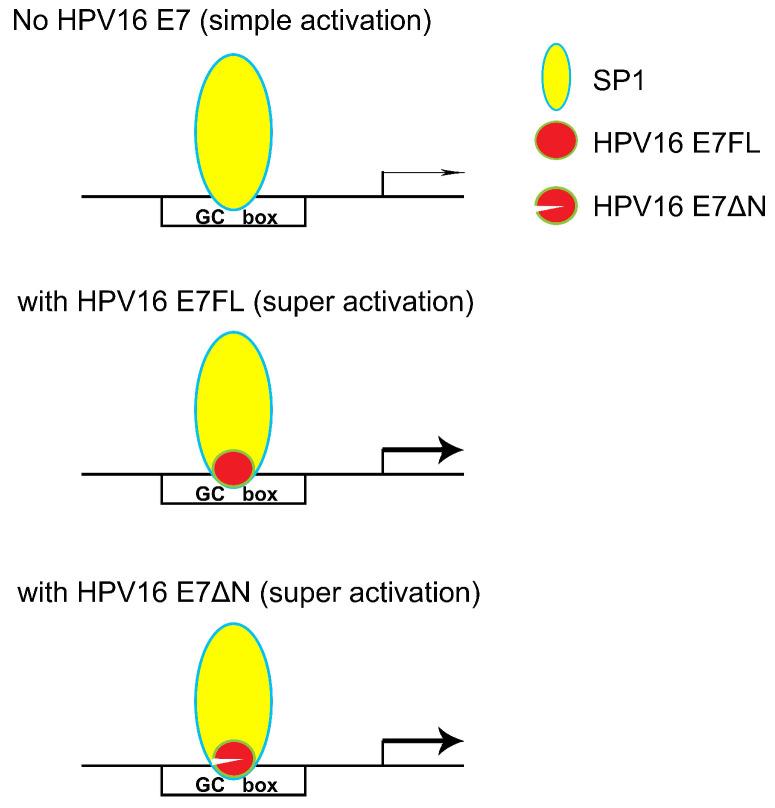
Model for HPV16 E7 involved in SP1-driven promoter transcription.

In our previous research, SP1 was found the major transcription factor involved in the MALAT1 transcription regulation 18. When hr-HPV infected, how SP1 and E7 together involved in the MALAT1 transcription regulation was concerned. Our preliminary results showed that both SP1 and E7 bound to MALAT1 promoter and form ternary complexes. The model in which E7 and SP1 regulated transcriptional activity of MALAT1 via GC box was shown in Figure 6. In non-HPV-infected cells, SP1 protein functioned as a simple activation transcription factor binding at GC box on the MALAT1 promoter 18. When hrHPV infections occurred, E7 recruited more SP1 and bound to the MALAT1 promoter forming super activation transcription complexes and increased the transcription. Meanwhile, E7 protein also displayed conformational transitions of dimer and monomer species, E7 dimer was very stable and resistant to thermal denaturation even in the presence of SDS 34, this conformational property was supposed to enable protein-protein recognition of the large number of celluar binding partners 34. In our experiments, two bands of complexes were observed, the slower migrating complexes detected by crosslinking assay likely represent dimer of E7 stacked on top of monomer bound to probe DNA (Figure 4C).

In addition, wild type E7 or E7ΔN both could increase cell growth, metastasis and invasion. E7ΔN contain a CKII site and a zinc finger domain. Our results were consistent with previous report. In fact, the replacement of the two Ser-residues of the CKII site with Ala could inhibit the transforming potential of E7. Furthermore, some mutations within the C-terminal Zinc-finger domain inhibit E7 transforming activity 35, 36 and impair the ability of E7 to immortalize human keratinocytes 31. These findings and our results suggested that CKII site and zinc finger domain of E7 played important role for the transforming activity.

It was mentioned that phosphorylated E7 by CKII enhanced the affinity of the viral protein for TBP, thus affected the activation of gene transcription mediated by TATA box 37, 38. Here, MALAT1 gene was TATA-less gene, therefore the zinc finger domain appeared to correspond for the transcription activation activity. In previous studies, it was reported that E7 zinc finger domain was involved in the E7 homodimer formation and responsible for the direct binding to target proteins such as HDAC, C-Jun and E2F1 3, 39, 40. Here in our research, SP1 was another target for E7. Another study revealed two separable transactivation domains of E7, the N terminus and C terminus of the protein 4. These data suggested that E7 itself might contribute to the transcriptional activation.

In summary, LncRNA MALAT1 was highly expressed in cervical cancer cells and was correlated with the metastasis of cervical cancer 13, 14, 41. MALAT1 was proposed to be a potential diagnostic marker. Here we demonstrated that viral E7 oncoprotein mediated transcriptional upregulation of MALAT1 by targeting the MALAT1 promoter, and provided a novel mechanism for E7 function. Our research helps the understanding of the association between HPV infection and the development of cancer.
